# α-Glucosidase Inhibitory Activity of Cycloartane-Type Triterpenes Isolated from Indonesian Stingless Bee Propolis and Their Structure–Activity Relationship

**DOI:** 10.3390/ph12030102

**Published:** 2019-07-01

**Authors:** Niken Pujirahayu, Debu Kumar Bhattacharjya, Toshisada Suzuki, Takeshi Katayama

**Affiliations:** 1 Laboratory of Biomass Chemistry, Faculty of Agriculture, Kagawa University, Kagawa 761-0795, Japan; 2 Department of Forestry, Faculty of Forestry and Environmental Sciences, Halu Oleo University, Kendari 93232, Indonesia; 3 Department of Biochemistry, Sher-e-Bangla Agricultural University, Dhaka 1207, Bangladesh

**Keywords:** cycloartane-type triterpenes, *Tetragonula sapiens* propolis, antioxidant activity, α-glucosidase inhibitory activity, structure-activity relationships

## Abstract

This study reports on the antioxidant activity and α-glucosidase inhibitory activity of five cycloartane-type triterpenes isolated from Indonesian stingless bee (*Tetragonula sapiens* Cockerell) propolis and their structure–activity relationships. The structure of the triterpenes was determined to include mangiferolic acid (**1**), Cycloartenol (**2**), ambonic acid (**3**), mangiferonic acid (**4**), and ambolic acid (**5**). The inhibitory test results of all isolated triterpenes against α-glucosidase showed a high potential for inhibitory activity with an IC_50_ range between 2.46 and 10.72 µM. Among the compounds tested, mangiferonic acid (4) was the strongest α-glucosidase inhibitor with IC_50_ 2.46 µM compared to the standard (–)-epicatechin (1991.1 µM), and also had antioxidant activities with IC_50_ values of 37.74 ± 6.55 µM. The study on the structure–activity relationships among the compounds showed that the ketone group at C-3 and the double bonds at C-24 and C-25 are needed to increase the α-glucosidase inhibitory activity. The carboxylic group at C-26 is also more important for increasing the inhibitory activity compared with the methyl group. This study provides an approach to help consider the structural requirements of cycloartane-type triterpenes from propolis as α-glucosidase inhibitors. An understanding of these requirements is deemed necessary to find a new type of α-glucosidase inhibitor from the cycloartane-type triterpenes or to improve those inhibitors that are known to help in the treatment of diabetes.

## 1. Introduction

Diabetes mellitus is a chronic disease caused by inherited and/or acquired deficiency in insulin production by the pancreas or by the ineffectiveness of the insulin produced. There are three main types of diabetes pathogeneses: Type 1 diabetes, also known as insulin-dependent, in which the pancreas fails to produce insulin, which is essential for survival. In type I diabetes, β-cells in the islets of Langerhans of the pancreas are damaged, and insulin is not secreted. Type 2 diabetes (known as non-insulin-dependent) results from the body’s inability to respond properly to the actions of insulin produced by the pancreas. Another type of diabetes is gestational diabetes mellitus (GDM) or hyperglycaemia in pregnancy. In type II diabetes, “insulin resistance” hyperglycemia is the result of inadequate production of insulin and the inability of the body to respond fully to insulin. Consequently, sugars flow into the blood and diabetes develops [1,2].

Postprandial hyperglycemia is a major risk factor in the development of type II diabetes [3]. The control of postprandial hyperglycemia is important in the treatment of diabetes mellitus. The most effective methods for preventing diabetes and hyperglycemia involve the control of glucose levels in the blood [4]. Sugar in the blood comes from the hydrolysis of carbohydrates and is catalyzed by digestive enzymes, such as α-glucosidase, which is secreted from the chorionic intestinal epithelium. α-Glucosidase inhibitors slow down the digestion process and absorption of carbohydrates by competitively blocking glucosidase activity. As a result, the peak concentration of postprandial blood glucose is reduced, and blood sugar levels are controlled [3,5].

Some α-glucosidase inhibitors, such as acarbose and voglibose, which were originally obtained from natural sources by fermentation, can effectively control blood glucose levels after food intake and have been used clinically in the treatment of diabetes mellitus [6]. However, their synthesis involves complicated multistep procedures. Also, they have been clinically associated with serious gastrointestinal side effects [3], such as flatulence and pain, nausea, skin reactions, abnormal liver function, and possible diarrhea [7]. Therefore, the search for inhibitors of α-glucosidase activity from natural products that do not cause undesirable effects on diabetics is essential.

Propolis is a non-timber forest product produced by honeybees. The bees collect resin from the crevices of bark and bud exudate of plants, chew the resin, add salivary enzymes, partially consume the resin to digest it, and then mix it with beeswax [8,9]. Some studies have proven that propolis has various biological functions, such as anti-microbial [10,11], anti-cancer [12,13], antioxidant, and anti-inflammatory activities [14,15].

Recent reports indicated that propolis mediates hypoglycemic effects of diabetes in patients and animal models [16]. Zhu et al. (2011) reported that Chinese and Brazilian propolis significantly prevented the progression of diabetes caused by Streptozotocin in mice and reduced oxidative stress in diabetic rats [17]. It is known that the primary botanical source of Brazilian propolis *is Baccharis dracunculifolia* (Asteraceae), and a prenylated p-coumaric acid is the main biologically active substance in this propolis, while Chinese propolis is a poplar type propolis, in which flavonoids, cinnamic acid, and its esters are the main active components. However, the effects of Chinese propolis on the inhibition of α-glucosidase and its inhibitory constituents are unclear.

Indonesian propolis has a different main compound from Brazilian propolis and Chinese propolis. In a previous study, we isolated and identified five cycloartane-type triterpenes as the main compounds from stingless bee *Tetragonula sapiens* propolis collected from Southeast Sulawesi, Indonesia, where its plant origin is *Mangifera indica* [18]. 

Several studies have reported that the following triterpenes have some biological activity, possibly as α-glucosidase inhibitors: corosolic acid, maslinic acid [19], betulinic acid, ursolic acid, and oleanolic acid [20,21]. However, reports of inhibition of cycloartane-type triterpenes on α-glucosidase are limited, and there have been no reports about the effects of their structure–activity relationships on the strength of α-glucosidase inhibition. Antioxidants are substances that can prevent, stop, or reduce oxidative damage; therefore, they are able to protect the human body from several diseases, such diabetes and the complications associated with this disease [22]. The combination of α-glucosidase inhibitors and antioxidants was recently shown to be effective on treating diabetes mellitus and preventing its development [23,24].

To find and design specific inhibitors of enzymes such as an α-glucosidase in the future, information and understanding of inhibition mechanisms and inhibitors’ structure–activity relationships are necessary. In this study, we report the antioxidant activity and phenolic content of three fractions of propolis: an ether fraction, an ethyl acetate fraction, and an aqueous fraction and the isolated compounds. The inhibitory activity of the isolated triterpenes, the mechanisms of their inhibition on α-glucosidase, and their structure–activity relationships are also reported.

## 2. Results

### 2.1. Extracts and Fractions Obtained from T. sapiens Propolis

Raw propolis P1 and P2 samples were extracted according to the method described in our previous study [18] to obtain Ethanol Extract of Propolis (EEP). EEP was then partitioned between diethyl ether, and ultrapure water, and the aqueous layer was extracted with ethyl acetate (EtOAc), P1 and P2 both gave three fractions as shown in Table 1.

### 2.2. Total Phenolic Content and Antioxidant Activity of T. sapiens Propolis

The phenolic content and DPPH radical scavenging activity of the three fractions (diethyl ether, ethyl acetate, and aqueous fractions) of propolis collected from two regions in Southeast Sulawesi, the South Konawe (P1) and Kendari (P2) were observed to vary (Table 2).

**Figure 1:**
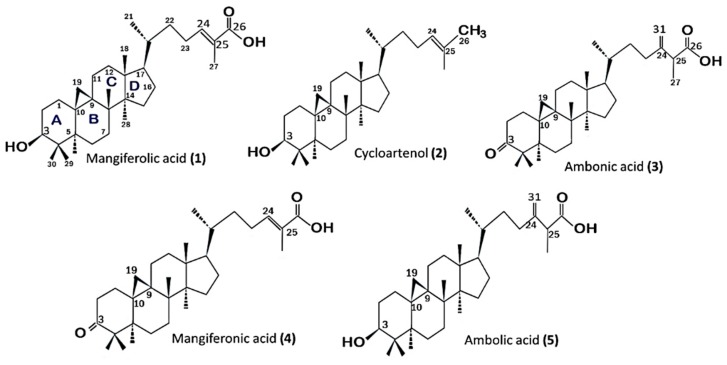
Structure of cycloartane-type triterpenes isolated from *T. sapiens* bee propolis in Southeast Sulawesi.

After a series of chromatographic-separation processes from ether fractions (P1 and P2) of EEP, five cycloartane-type triterpenes were obtained (Figure 1). Then the five isolated compounds were tested for antioxidant activity and the results are shown in Table 3.

### 2.3. Inhibitory Activity and Kinetic Inhibition of Cycloartane-Type Triterpenes Isolated from T. sapiens Bee Propolis on α-Glucosidases

Our previous paper [18] reported that five compounds were isolated from Ethanol Extract of Propolis (EEP), and their structures were determined to be mangiferolic acid (**1**) [25,26,27], cycloartenol (**2**) [25,27], ambonic acid (**3**), mangiferonic acid (**4**), and ambolic acid (**5**) [25,26,28], which are all cycloartane-type triterpenes (Figure 1). 

The inhibitory effects of the isolated compounds on α-glucosidases from yeast (*Saccharomyces cerevisiae*) and rat small intestine were examined, and the results are shown in Table 4.

**Figure 2:**
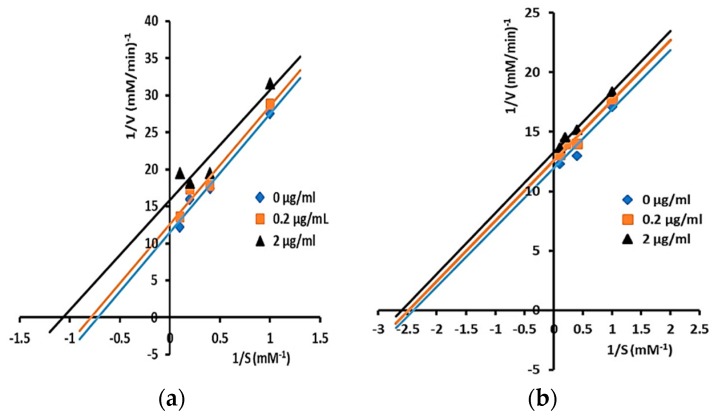
Lineweaver–Burk plots for kinetic analysis of α-glucosidase inhibition by isolated triterpenes: (**a**) compound **1** and; (**b**) compound **2** at varying concentrations (showing uncompetitive inhibition). The concentration of p-NPG was measured as a substrate in the absence or presence of inhibitors at different concentrations against α-glucosidase. All values are means ± standard errors (*n* = 3).

**Figure 3:**
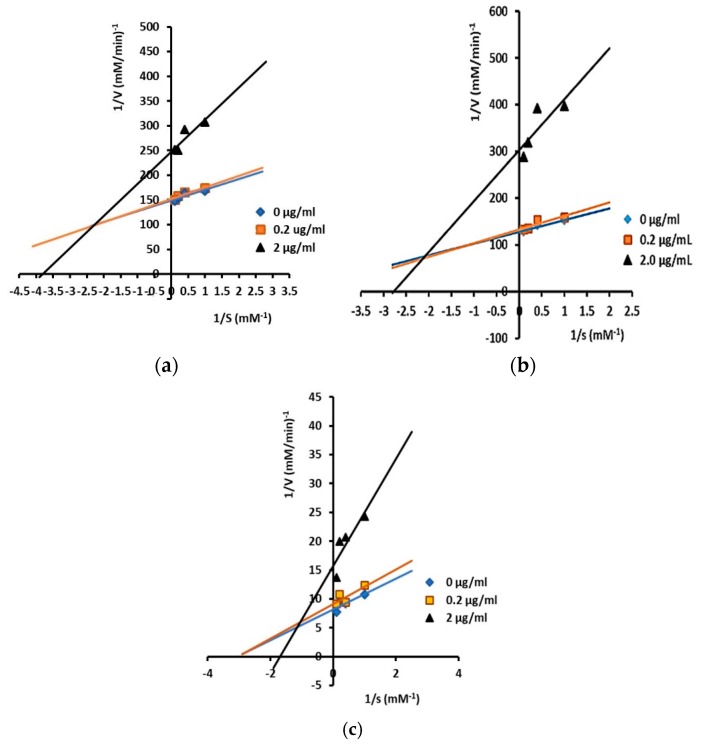
Lineweaver–Burk plots for kinetic analysis of α-glucosidase inhibition by isolated triterpenes: (**a**) compound **3**; (**b**) compound **4**; and (**c**) compound **5** at varying concentrations (showing mixed inhibition). The concentration of p-NPG was measured as a substrate in the absence or presence of inhibitors at different concentrations against α-glucosidase. All values are means ± standard errors (*n* = 3).

Inhibitory mechanisms of isolated triterpenes against α-glucosidase at some concentrations (0, 0.2, and 2 µg/mL) were analyzed by the Lineweaver–Burk plot (Figure 2 and Figure 3).

### 2.4. Structure–Activity Relationship of the Cycloartane-Type Triterpenes Isolated from T. sapiens Propolis and Their Inhibitory Activity against α-Glucosidase

**Figure 4:**
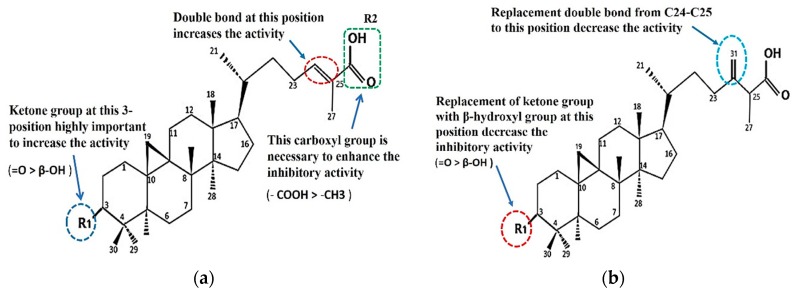
Structure–activity relationship for α-glucosidase inhibition of cycloartane-type triterpenes **1**, **2**, **4** (**a**) and **3**, **5** (**b**) from *T. sapiens* propolis.

The comparison of the structure and inhibitory activity against α-glucosidase between the isolated compounds showed that functional groups of cycloartane-type triterpenes had a significant impact on the inhibition (Figure 4a,b).

## 3. Discussion

### 3.1. Phenolic Content and Antioxidant Properties of T. sapiens Propolis

Table 1 shows that the range of propolis phenolic content from two collection sites was between 13.9 and 37.2 μg gallic acid equivalent/mg sample. The ethyl acetate fraction of propolis from Kendari district (P2) had a higher total phenolic content of 37.2 μg/mg compared with the phenolic content of P1, while the lowest content was in the aqueous fraction of P2.

The range of DPPH radial scavenging activity (IC_50_ value) of P1 ranged from 25.0 to 84.3 μg/L, and the aqueous fraction had the highest IC_50_ of 19.7 μg/L compared with the other two fractions. In the case of P2, the antioxidant value of propolis ranged from 25.4 to 84.3 μg/mL, and the ether-soluble fraction had the highest IC_50_ value of 25.4 μg/mL. This value shows moderate–high activity in comparison with the previously reported antioxidant activity of Indonesian stingless bees propolis from other site collections that had IC_50_ values between 68.9 and 4162.6 μg mL^−1^ [29].

Therapeutic properties of propolis are often associated with the presence of polyphenols. However, large heterogeneity has been found in their chemical composition. As a result, various components, and not only phenolic compounds, can have biological attributes. Several studies have reported the biological properties of propolis were associated with diterpenes and triterpenes as compounds responsible for their pharmacological properties [30,31]. In this study, triterpenes were the main compound of *T. sapiens* propolis collected from two different regions.

Antioxidants have been reported to decrease some of the damaging effects of free radicals on the pancreas, resulting in the restoration of insulin functioning and subsequent lowering of blood glucose concentration, and may retard associated complications [32]. An ideal anti-diabetes compound should exhibit the activities of α-glucosidase inhibitors and properties of antioxidants [23,24]. Cycloartane-type triterpenes isolated from *T. sapiens* showed moderate-high antioxidant activity (Table 3). Mangiferonic acid (1) had an antioxidant activity greater than the original ether-soluble extract, therefore it can be assumed that the compound is responsible for the antioxidant activity in the extract of propolis (P2). 

### 3.2. Inhibitory Activity and Kinetic Inhibition of Cycloartane-Type Triterpenes Isolated from T. sapiens Bee Propolis on α-Glucosidases

Cycloartane belongs to tetracyclic triterpenoids, which contain A, B, C, and D rings and 30 carbon atoms (Figure 1). Furthermore, all isolated compounds also contain a cyclopropane ring. Cycloartanes are mainly distributed in Leguminosae, Passifloraceae (*Passiflora edulis*), Ranunculaceae (*Cimicifuga racemos*) [33], Anacardiaceae (*M. indica*) [28], and Euphorbiaceae [34]. In comparison to pentacyclic triterpenes, the distribution of tetracyclic triterpenes in medicinal plants is not wide-spread, and they often taste bitter [33]. It is thought that these components cause the taste of this *T. sapiens* propolis to be rather bitter and sour.

All isolated compounds showed a high potential inhibitory activity against the α-glucosidase from the yeast (*S. cerevisiae*) with an IC_50_ range between 2.46 and 10.72 µM (1.12‒4.56 µg/mL) compared to the standard (−)-epicatechin concentration of 1991.1 ± 89.9 µM (577.95 µg/mL). The IC_50_ of (−)-epicatechin was similar to previously reported value of 510 µg/mL [35]. Among the isolated compounds tested, mangiferonic acid (**4**) was shown to be the strongest α-glucosidase inhibitor with IC_50_ 2.46 µM (1.12 µg/mL). 

(−)-Epicatechin showed inhibitory activity against α-glucosidase from the yeast (*S. cerevisiae*), but it had no inhibitory activity against that from the rat small intestine. In contrast, acarbose and voglibose showed very high inhibitory activity against α-glucosidase from the rat small intestine but no activity at all against that from the yeast. Similarly, previous studies [19,35,36] reported that epicatechin showed inhibitory activity on α-glucosidase from yeast but not against mammalian α-glucosidase due to differences in the molecular recognition of the target-binding site of the enzyme [37]. (–)-epicatechin is used as a standard in this study because it is a flavonoid that has antihyperglycemic and insulinogenic activity [38] and had a high inhibitory value on α-glucosidase, around 96% [3,39]. 

Acarbose and Voglibose are standards commonly used in an α-glucosidase inhibition assay. Many studies reported that acarbose or voglibose could prolong the duration of carbohydrate absorption and flatten blood glucose concentration over time curve. Therefore, acarbose and voglibose have been used as first-line drugs in the treatment of Type 2 diabetes, which is not controlled through diet alone, [19,40]. Acarbose has been used clinically to prevent postprandial glycemia hypertension for many years, and there have been several reports showing that it is the ability to limit or prevent postprandial hyperglycemia in human and mice as well [41,42]. However, it was reported in some studies that acarbose weakly inhibited yeast α-glucosidase [32,43] and some showed no inhibition [19,40], but strongly inhibited α-glucosidase from mammals such as rat intestines [21].

Modes of inhibition of isolated triterpenes were determined by analysis of Lineweaver–Burk plots and calculated by Michaelis–Menten kinetics. Compounds **1** and **2** displayed uncompetitive inhibition as shown by the straight parallel lines in the plot of 1/V versus 1/[S] (Figure 2a,b), while compounds **3**, **4**, and **5** showed mixed inhibition against α-glucosidase (Figure 3a–c). The kinetic analysis of compounds **1** and **2** showed that both V_max_ and Km decreased in the presence of an increasing concentration of inhibitors, with Ki values of **1** and **2** being 6.04 and 10.27 µM, respectively. Uncompetitive inhibitors do not bind to the free enzyme site but only to an enzyme–substrate complex. When the inhibitor concentration increases, more enzymes will be converted into unproductive enzyme–substrate inhibitors (E.S.I). Thus, both the Km and the V_max_ values are decreased by the same amount; this is the exact opposite of the competitive case [44].

As illustrated in Figure 3, compounds **3**, **4**, and **5** showed mixed-type inhibition against α-glucosidase. A compound will behave as a mixture of a competitive and a non-competitive inhibitor or will show mixed inhibition when it can bind both to the free sites of enzymes (competitive) and the enzyme–substrate sites (uncompetitive) [44], which are indicated by increased Km values and decreased V_max_ values.

Furthermore, the values of inhibiting constants (Ki and Ki’) of compounds **3**, **4**, and **5** showed that they have a higher affinity for free enzymes than for enzyme–substrate, as the value of Ki was smaller than Ki’ in compound **3** (Ki = 2.11 µM and Ki’ = 6.42 µM), compound **4** (Ki = 0.58 µM and Ki’ = 3.23 µM), and compound **5** (Ki = 1.71 µM and Ki’ = 4.58 µM).

For mixed inhibition, the inhibitor would be still effective at lower concentrations compared to a competitive inhibitor like acarbose. It was reported that in competitive inhibitors such as acarbose, higher inhibitor concentrations are needed for higher carbohydrate food intake to show the same inhibitory effect [45,46].

### 3.3. Structure–Activity Relationship of the Cycloartane-Type Triterpenes Isolated from T. sapiens Propolis and Their Inhibitory Activity against α-Glucosidase

The comparison of the structure and the inhibitory activity against α-glucosidase between the five isolated compounds showed that the functional groups of cycloartane-type triterpenes had significant impacts on the inhibition. The ketone group at C-3 and the carboxyl group at C-26 are important to enhance the inhibitory activity. The IC_50_ of ambonic acid (**3**) is almost the same as that of **4**. We conclude that the substituents at C-3 are important components of the cycloartane skeleton that can strengthen the inhibitory activity of α-glucosidase in the order C =O > β-OH and that the substituents at C-26 are also important components in the order of –COOH > –CH_3._ As a result of the isolated compounds, cycloartenol (**2**) has the lowest IC_50_, where the carboxyl group on C-26 is replaced by a methyl group (Figure 2). If a substituent in C-3 replaces =O with β-OH, the activity will decrease by almost 1.5-fold, (from 2.46 (compound **4**) to 5.52 µM (compound **1**), but if the double bond in C-24–C-25 shifts to C-31, the activity will decreases by 0.3-fold (from 2.46 (compound **4**) to 3.01 µM (compound **3**) (Figure 4 and Table 2)).

Interestingly, the presence of the terminal double bond with methylene (=CH_2_) on C-31 can also increase the activity of **5** compared to that of **1**, but it slightly reduces the activity of **3** when compared to **4**. The structure of compound **3** is similar to that of **5**; it is different only in the substituents in C-3. However, this difference in constituents also results in differences in the strength of the α-glucosidase inhibitory in both. At **3**, the substituent at C-3 is a ketone (=O), and if replaced with β-OH, as it is in **5**, the inhibiting activity is reduced to half-fold (from 3.01 µM to 4.31 µM).

Nguyen et al. (2016) [47] reported that mangiferonic acid (**4**) isolated from bark of *Mangifera mekongensis* in Vietnam had the highest inhibitory activity against α-glucosidase from *Saccharomyces cerevises* compared with the other isolates, mangiferolic acid (**1**) and ambolic acid (**5**) (where C-3 is β-OH); however, the inhibitory activity and their structure–activity relationships were not reported. Another cycloartane-type triterpene isolated from *Schisandra chinensis*, 24-methylenecycloartenone, which also contains a ketone group at C-3, is reported to have high inhibitory activity against α-glucosidase with an IC_50_ value of 2.36 μM [5], which is almost the same as that found for mangiferonic acid (**4**) in this study. 

This study demonstrated that the five cycloartane-type triterpenes isolated from *T. sapiens* propolis as the main components have high inhibitory activity against α-glucosidase. 

## 4. Materials and Methods

### 4.1. Sample Preparation

Samples of raw propolis P1 and P2 were extracted and isolated according to methods described in previous studies [18] to obtain the Ethanol Extract of Propolis (EEP). EEP was then partitioned between diethyl ether (50 mL) and ultrapure water (50 mL) with a separatory funnel to give an ether-soluble fraction and an aqueous layer. The latter was again extracted with ethyl acetate (EtOAc), and the EtOAc-soluble fraction and the aqueous fraction were obtained (Table 1). According to fractionation results, Ether fraction gave the highest yield in this propolis. As such, the isolation of the compounds contained in this fraction was carried out using a series of chromatographic separation experiments as was done in the previous report and obtained 5 isolated compounds [18] used in this study. Each sample (fractions and isolated compounds) was prepared as much as 1 mg for use in subsequent assay (total phenolic content, antioxidant activity, the inhibitory activity on α-glucosidase and kinetics enzyme).

### 4.2. Total Phenolic Content 

Total phenol contents in propolis were determined by the Folin–Ciocaltecu method according to Suzuki et al. (2016) [48] with some modifications. The tree fractions (ether fraction, ethyl acetate fraction, and aqueous fraction from the EEP) were dissolved in methanol to make a 100 ppm solution. Each of the test solutions (0.2 mL) was added to a mixture of 0.2 mL of the Folin–Ciocalteceu reagent (Nacalai Tescue Inc, Kyoto, Japan) diluted two-fold with water and 0.2 mL of 10% (*w*/*v*) sodium carbonate solution. The resulting mixture were shaken vigorously and left at room temperature for 20 min. Color development at 760 nm was measured with an Epoch microplate reader (Biotech, Tokyo, Japan). The calibration curve was obtained from various concentration of gallic acid. Total phenolic contents were expressed as µg/mg (gallic acid equivalents).

### 4.3. DPPH Free Radical Scavenging Activity

The antioxidative activities of the fractions were assayed for the scavenging of 2,2-diphenyl-1-picrylhydrazyl (DPPH) (Sigma-Aldrich, Darmstadt, Germany). Free radicals were assayed using a previously reported method [48] with some modifications. The fractions of EEP, isolated compounds and Trolox (Sigma-Aldrich, Darmstadt, Germany) were dissolved in methanol to make a 50 ppm solution. Each of the test sample solutions (0, 20, 40, 60, 80, and 100 µL) were added into a mixture (0.9 mL) of 0.4 mM DPPH solution, 20% methanol aqueous solution, and 0.2 M MES (2-(N-morpholino) ethanesulfonic acid) (Dojindo, Wako Junyaku Kogyo Co., Ltd., Osaka, Japan) buffer solution (1:1:1). The resulting mixtures were shaken on a vortex mixer and allowed to stand for 20 min. The absorbance of the remaining DPPH was measured with a microplate reader (Biotech, Tokyo, Japan) at 520 nm. The percentage of DPPH radical inhibition by each sample was expressed as the percentage inhibition relative to the control, and the IC_50_ value (50% inhibition concentration) was calculated by the following formula:

(1) $$DPPH\;free\;radical\;scavenging\;\left(\%\right)=\left(1-\frac{A1}{A0}\;\right)\times \;100\;\;\;\;\;\;\;\;\;$$

where *A*0 is the absorbance of the mixture without a sample, and *A*1 is the absorbance of the mixture with a sample after 20 min. The inhibitory concentration that results in 50% scavenging of DPPH radicals (IC_50_) was estimated based on the plot of inhibition versus the final concentration of the propolis samples. The results are expressed in µg Trolox Equivalents (TE) per mL.

### 4.4. Inhibitory Activity Assay for Yeast α-Glucosidase

The inhibitory activity of α-glucosidase from *Saccharomyces cerevisiae* (Sigma-Aldrich, Tokyo, Japan) of the isolation compounds was determined using a previously reported procedure with modifications [35,36]. p-Nitrophenyl α-glucopyranoside (pNPG) (Sigma-Aldrich, Tokyo, Japan) was used as a substrate. Other chemicals used were of analytical grade. Quickly, 20 µL of isolated compounds at various concentrations (0.1 µg/mL–10 µg/mL or 0.2 µM–23.5 µM) and positive controls (−)-epicatechin (Sigma-Aldrich, Tokyo, Japan), acarbose, and voglibose (Wako Pure Chemical Industries. Ltd., Tokyo, Japan) (0.5, 1.0, and 5.0 mg dissolved in methanol and water 1:9 *v*/*v*) were added to a 96-well plate with 40 µL of α-glucosidase solution (0.1 U/mL) in phosphate buffer (pH 7.0) and were mixed and incubated at 37 °C for 5 min. Then, 40 µL of 3 mM pNPG solution was added, and the mixture was incubated at 37 °C for 20 min. The reaction was stopped by adding a solution of Na_2_CO_3_ (0.1 M, 200 µL), and the absorbance (at 405 nm) of the resulting solution was measured via an Epoch microplate reader (Biotech, Tokyo, Japan). All experiments were performed in three independent replicates. The inhibition of isolated compounds on α-glucosidase was calculated as follows:

(2) $$Inhibition\;\left(\%\right)=\left(1-\frac{A1}{A0}\right)\times 100$$

where *A*0 is the absorbance of the control, and *A*1 is the absorbance of the propolis samples. The IC_50_ values were determined as the half-maximal inhibitory concentration of the inhibitor (isolated compound) which provides 50% inhibition.

### 4.5. Inhibitory Activity Assay for Rat Intestinal α-Glucosidase 

In a centrifuge tube at 4 °C, 200 mg of rat intestine acetone granules (Sigma-Aldrich, Tokyo, Japan) was placed in 8 mL of physiological saline (0.9 g of NaCl was dissolved in 100 mL pure water). The resulting suspension was homogenized with sonication equipment (Branson, Danbury, CT, USA) for 60 s (ultrasonic treatment cycles: 50, output control: 5) in an ice bath. The resulting homogenate was centrifuged (14,400× *g*, −21 °C for 20 min) with a high-speed refrigerated centrifuge (Himac CR21N, Hitachi Koki Co, Ltd., Tokyo, Japan). The resulting supernatant was used as an enzyme preparation, and stored at −30 °C until use [35]. Then the measurement of inhibitory activity of isolated compounds against α-glucosidase from the rat small intestine followed the method used to measure the activity of yeast enzymes (*S. cerevisiae*). The concentration of the isolated compounds used is 0.1–10 µg/mL (0.2–23 µM).

### 4.6. Determination of Inhibitory Type by Kinetic Analysis

The inhibition activity of isolated triterpenes against α-glucosidase was measured with different concentrations (0, 1, 2.5, 5, and 10 mM) of p-NPG as a substrate in the presence or absence of different concentrations (0, 0.2, and 2 µg/mL) of the compounds. The mode of inhibition was then determined by Lineweaver–Burk plot analysis, which was calculated using Michaelis–Menten kinetics. Inhibition constants (Ki) were determined by analysis of Dixon plots and plots of the Y-intercept of the Lineweaver–Burk plot versus the inhibitor [44,49]. All data are expressed as means ± standard errors of triplicate determinations.

## 5. Conclusions

Five cycloartane-type triterpenes, namely mangiferolic acid, cycloartenol, ambonic acid, mangiferonic acid, and ambolic acid, isolated from *Tetragonula sapiens* propolis were shown to have high inhibition activity against α-glucosidase from *Saccharomyces cerevisiae*, with mangiferonic acid being the most active inhibitor. According to an enzyme kinetics study, the inhibition types of the isolated cycloartane-type triterpenes were uncompetitive inhibition and mixed inhibition. The inhibitory activity of cycloartane-type triterpenes against α-glucosidase depends on the substituents located on C3, the double bonds on C24–C25, and a carboxyl group on C26. This is the first report of inhibitory activity of cycloartane-type triterpenes isolated from propolis against α-glucosidase. This study provides an approach to consider the structural requirements of cycloartane-type triterpenes from propolis as α-glucosidase inhibitors. An understanding of these requirements is needed to be able to find, improve, or design new types of cycloartane-type α-glucosidase inhibitors from triterpene to help in the treatment of diabetes. Some compounds (mangiferolic acid, mangiferonic acid and ambonic acid) also have moderate-high activity where antioxidant activity can help accelerate healing and prevent complications in diabetics, which makes this compound have more ideal potential as an anti-diabetes compound.

## Figures and Tables

**Table 1:** Extracts and Fractions obtained from *T. sapiens* propolis.

No	Extract & Fractions	Weigh/Yield (g)	%
1.	Raw propolis of P1	10.00	
	Ethanol Extract of Propolis (EEP) of P1	5.66	56.6
	Diethyl ether fr. of EEP P1	4.87	86.0
	Ethyl acetate (EtOAc) fr. of EEP P1	0.09	1.59
	Aqueous fr. of EEP P1	0.56	9.89
2.	Raw propolis of P2	15.00	
	EEP of P2	9.97	66.5
	Diethyl ether fr. of EEP P2	7.35	73.7
	Ethyl acetate (EtOAc) fr. of EEP P2	0.51	5.11
	Aqueous fr. of EEP P2	0.57	5.71

**Table 2:** Phenolic content and antioxidant activity of *T. sapiens* propolis collected from two regions (P1 and P2).

Sample	Phenolic Acid (GAE) (µg/mg) ^a^	Antioxidant Activity IC_50_ (µg/mL) ^a^
P1 ether Fraction	16.7 ± 0.218	64.9 ± 4.30
P1 EtOAc fraction	21.5 ± 0.271	25.0 ± 3.03
P1 aqueous fraction	24.4 ± 0.612	19.7 ± 0.229
P2 ether Fraction	32.4 ± 0.311	25.4 ± 3.78
P2 EtOAc Fraction	37.2 ± 0.468	32.44 ± 6.32
P2 aqueous fraction	13.9 ± 0.198	84.3 ± 9.71
Trolox	-	6.76 ± 0.395

^a^ The values are the means ± SEs, *n* = 3.

**Table 3:** Antioxidant activity of isolated compounds from the ether fraction of *T. sapiens* propolis.

Compounds	DPPH	
	IC_50_ (µg/mL) ^a^	IC_50_ (µM) ^a^
Mangiferolic acid (1)	12.38 ± 3.0	27.11 ± 6.57
Cycloartenol (2)	120.21 ± 11.2	282.18 ± 26.3
Ambonic acid (3)	42.45 ± 4.5	90.57 ± 9.60
Mangiferonic acid (4)	17.16 ± 2.98	37.74 ± 6.55
Ambolic acid (5)	132.30 ± 13.9	281.30 ± 29.5
Trolox ^b^	7.17 ± 0.13	28.62 ± 0.52

^a^ The values are the means ± SEs, *n* = 3, ^b^ Positive control.

**Table 4:** IC_50_ values and inhibition mode of the cycloartane-type triterpenes isolated from Indonesian *T. sapiens* propolis against α-glucosidases.

Compounds	Yield (mg)	IC_50_		Inhibition Mode
		*S. cerevisiae* (µM) ^a^	Rat Small Intestine (µM) ^a^	
Mangiferolic acid (**1**)	20.6	5.52 ± 0.04	ND	Uncompetitive
Cycloartenol (**2**)	5.9	10.72 ± 0.28	ND	Uncompetitive
Ambonic acid (**3**)	42.4	3.01 ± 1.26	ND	Mixed inhibition
Mangiferonic acid (**4**)	68.5	2.46 ± 0.70	ND	Mixed inhibition
Ambolic acid (**5**)	24.8	4.31 ± 0.04	ND	Mixed inhibition
(–)-Epicatechin ^c^		1991.1 ± 89.9	ND	
Acarbose ^c^		ND ^b^	208.95 ± 0.96	
Voglibose ^c^		ND	78.57 ± 1.27	

^a^ The values are the means ± SEs, *n* = 3; ^b^ ND: not detected; ^c^ positive control.
